# Optimization of Stress-Based Microfluidic Testing for Methicillin Resistance in *Staphylococcus* *aureus* Strains

**DOI:** 10.3390/diagnostics8020024

**Published:** 2018-04-17

**Authors:** Maxim Kalashnikov, Jean C. Lee, Alexis F. Sauer-Budge

**Affiliations:** 1 Center for Manufacturing Innovation, Fraunhofer USA, Brookline, MA 02446, USA; mkalashnikov@fraunhofer.org; 2 Division of Infectious Diseases, Department of Medicine, Brigham and Women’s Hospital and Harvard Medical School, Boston, MA 02115, USA; jclee@rics.bwh.harvard.edu; 3 Department of Biomedical Engineering, Boston University, Boston, MA 02215, USA; 4 Exponent, Inc., Natick, MA 01760, USA

**Keywords:** antibiotic susceptibility testing, *Staphylococcus aureus*, MRSA, microfluidic chip, fluid shear, rapid diagnostics

## Abstract

The rapid evolution of antibiotic resistance in bacterial pathogens is driving the development of innovative, rapid antibiotic susceptibility testing (AST) tools as a way to provide more targeted and timely antibiotic treatment. We have previously presented a stress-based microfluidic method for the rapid determination of antibiotic susceptibility in methicillin-susceptible *Staphylococcus aureus* (MSSA) and methicillin-resistant *Staphylococcus aureus* (MRSA). In this method, stress is used to potentiate the action of antibiotics, and cell death is measured as a proxy for susceptibility. The method allows antibiotic susceptibility to be determined within an hour from the start of the antibiotic introduction. However, the relatively low dynamic range of the signal (2–10% cell response) even with high antibiotic concentrations (10–50 µg/mL) left room for the method’s optimization. We have conducted studies in which we varied the flow patterns, the media composition, and the antibiotic concentration to increase the cell death response and concordantly decrease the required antibiotic concentration down to 1–3 µg/mL, in accordance with the Clinical and Laboratory Standards Institute’s (CLSI) guidelines for AST breakpoint concentrations.

## 1. Introduction

The rapid evolution of antibiotic resistance in bacterial pathogens [1] has spurred a response at the national level: the United States’ initiative on combating antibiotic resistance [2]. This initiative calls for the development of rapid antibiotic susceptibility testing (AST) tools as a way to make antibiotic treatment more targeted and timely, thus increasing patient survival, safeguarding antibiotics, and reducing economic costs. One of the clinical conditions requiring rapid AST is bacteremia. Bacteremia is a clinical condition associated with the presence of pathogenic bacteria in otherwise sterile blood. This condition is associated with a 28–50% mortality rate due to septic shock [3]. Additionally, mortality increases 7% for each hour of improper or delayed antibiotic treatment [4]. Because of this, broad-spectrum antibiotics are prescribed as an empirical treatment; however, prescription can be inadequate in as many as 50% of the cases [5].

*Staphylococcus aureus* alone is associated with almost 10% of bloodstream infections [5], whereas methicillin-resistant *Staphylococcus aureus* (MRSA) is a major health concern associated with 8% of hospital-acquired infections in the US [6]. As such, it was identified as a serious threat by the CDC (Center for Disease Control, Atlanta, GA, USA) amongst 18 pathogen threats in 2013 [7]. Traditionally, antibiotic susceptibility is determined either by broth dilution or disk diffusion techniques [8]. These tests rely on the evaluation of bacterial growth in the presence of varying concentrations of antibiotic. The time required for growth of clinical samples to detectable limits for identification tests as well as the additional time needed for AST are bottlenecks in the traditional workflow for determining the susceptibilities of culturable clinical samples. Bacterial levels as low as 10–100 CFU (colony-forming units) per milliliter of infected blood are the major cause for the delay in appropriate therapy [9,10]. Standard AST protocols require bacteria to propagate until there are about 10^8^ CFU/mL prior to identification and AST (ca. 20 doublings or ≥12 h). 

Rapid susceptibility testing can be broadly divided into two categories: genetic and phenotypic. Genetic methods, such as PCR (polymerase chain reaction), provide fast (within 2 h after primary colonies are isolated) identification of strains carrying specific antibiotic resistance genes [11], such as the *mecA* gene for MRSA strains [12]. These methods are useful for screening, but they may fail in cases where newly acquired antibiotic resistance is independent of target gene expression. Whole genome sequencing [13] and transcriptomics [14] are becoming extremely useful tools in epidemiological studies during clinical outbreaks and can provide future specific targets for molecular testing, but due to required sample sizes and time to results, have limited AST utility. For example, in a previous study [14], it took 17 h to determine the RNA hybridization profile of laboratory-evolved *Escherichia coli*, catching snapshots of bacterial gene expression from 100,000 bacteria. One approach to accelerating phenotypic AST is focused on miniaturizing the standard process of bacterial growth and combining it with microscopy for micro-susceptibility testing [15]. The combination of confining bacteria to small volumes (microfluidics) and the direct or indirect microscopic observation of their growth has led to a significant reduction in AST time [16]. Alternative antibiotic susceptibility markers can provide even faster susceptibility readings. These alternative markers include fluorescence indicators of bacterial cell death with [17,18,19] or without [20] stress, monitoring changes in bacterial cell morphology in response to antibiotics [21,22], or measuring the change in bacterial vibrations [23]. 

We previously developed a stress-based microfluidic AST for MSSA/MRSA [18]. This rapid AST is based on monitoring the death of bacteria under mechanical and chemical stresses in the presence of antibiotics. Briefly, the bacteria are immobilized inside the channel of the microfluidic device on a chemically-activated glass channel floor. The experimental media (fluorescent dead cell stain, chemical stressor, and bacterial culture media) are flowed over the immobilized bacteria, delivering chemical components and providing mechanical shear stress. Bacterial fluorescence associated with dead bacteria is monitored as a function of time. Susceptible and resistant bacterial strains are separated based on the number of fluorescing cells. The method correctly identified the susceptibility profiles of 18 *Staphylococcus aureus* strains. Two significant limitations of the method remained unresolved: (1) obtaining the correct susceptibility assignment required a high antibiotic concentration (10 or 50 µg/mL), while clinically relevant concentrations are 2 µg/mL [8]; and (2) the cell death response for MSSA varied significantly between replicate experiments (2–10%). Thus, an increased response level and reduced antibiotic concentrations were desired for further advancement of the method. In the present work, we enhanced the cell death response of susceptible bacteria by determining the importance of and optimizing the following experimental parameters: temperature, media composition, amount of applied stress, and antibiotic concentration. 

## 2. Materials and Methods

### 2.1. Microfluidic Flow Cell

**Figure 1:**
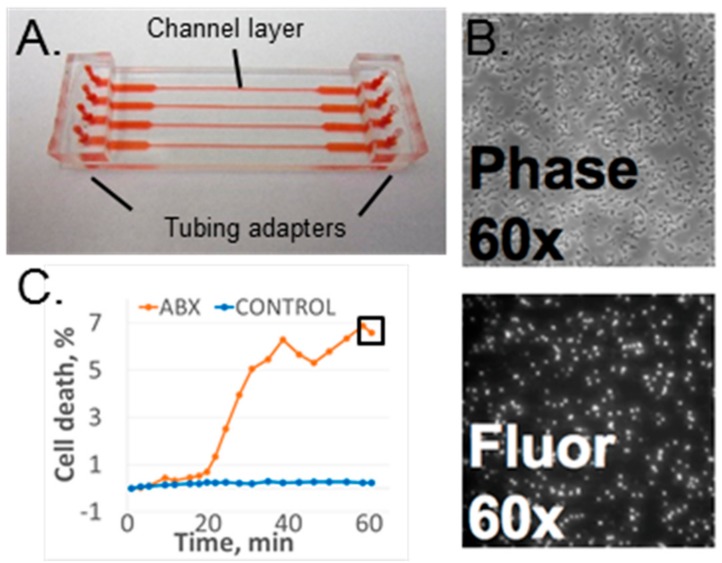
(**A**) Microfluidic antibiotic susceptibility testing (AST) device. Red shows filled channels. (**B**) Phase contrast and fluorescence images of susceptible *S. aureus* after 60 min of oxacillin exposure. (**C**) Data sample of time-dependent response for a susceptible strain with (ABX) and without antibiotic (control). The black square shows the 60-min time point.

The microfluidic flow device used for these experiments was the four-channel design used in our initial studies [16]; however, the device was assembled using our most recent published protocol [19]. Each device consisted of a polydimethylsiloxane (PDMS) (Sylgard^®^ 184, Dow Corning, Auburn, MI, USA) channel layer chemically bonded to a SuperEpoxy2 glass slide (ArrayIt Corp., Sunnyvale, CA, USA) to form an enclosed channel layer. The top side of the PDMS channel layer was bonded to macrofluidic PDMS adapters to connect input and output tubing. The channel layer was molded from PDMS using a standard molding process [24]. The inverse of the channel pattern was created on the metal mold surface using an ultraprecision milling machine (UPM-0005, Fraunhofer-IPT, Aachen, Germany). The PDMS and curing agent mixture (10:1) was cast over the mold and then cross-linked at 100 °C for 45 min. Note that the higher temperature of PDMS curing led to smaller channel dimensions than in the original publication [18]. Each channel was 135 µm deep, and its width varied from 370 µm in the narrow portion of the channel to 2.5 mm in the wide portion of the channel. The channel layer’s outer dimensions were 25 × 75 mm to match those of a standard #1 microscope slide. Instead of mechanical sealing, plasma-assisted chemical bonding was used to assemble the device [19]. The PDMS channel layer was oxygen-activated with plasma and thermally bonded to the epoxide-functionalized glass slide at 100 °C for 30 min. Both the top surface of the channel layer and the bottom surface of the tubing adapters were oxygen-plasma-activated for PDMS–PDMS bonding. The fully assembled device is shown in Figure 1A. Note that the epoxy glass slide is fully covered by the channel layer and cannot be seen in the figure. 

### 2.2. Experimental Setup

#### 2.2.1. Bacteria and Media

The bacterial samples were prepared and introduced into the device according to our previously reported protocol [17]. An overnight bacterial culture was inoculated into 1 mL of Mueller Hinton (MH) broth containing 2% NaCl (MHS). Bacteria were grown to log phase during a 2-h incubation at 37 °C with shaking at 300 rpm. A 40-µL bacterial suspension was loaded into each channel, and the assembly was centrifuged for 1 min at 1200 rpm. The media composition for each experiment is specified below, and all contained MH broth, the chemical stressor lysostaphin (Cell Sciences, Inc., Canton, MA, USA), oxacillin salt (Sigma Aldrich, Inc., St. Louis, MO, USA) and the dead cell fluorescent stain SYTOX Orange (Molecular Probes, ThermoFisher, Inc., Waltham, MA, USA). The assembled microfluidic device was connected to an automated liquid pump for media flow control (PHD ULTRA pump, Harvard Apparatus, Holliston, MA, USA).

#### 2.2.2. Imaging Hardware

The whole assembly was fixed to a 3-axis (XYZ) controlled stage (H117, Prior Scientific Inc., Rockland, MA, USA, H122R, Prior Scientific Inc., Rockland, MA, USA) of an Olympus IX-70 inverted fluorescence microscope (Olympus America Inc., Chelmsford, MA, USA). The phase contrast and fluorescence images of the bacteria were collected through a 60× microscope objective (LCPlanFl, Olympus America Inc., Chelmsford, MA, USA). Both the excitation and emission light passed through a filter cube (U-MWIG, 520–550 nm excitation, >580 nm emission, Olympus America Inc., Chelmsford, MA, USA). The collected image was limited to a 250-µm by 250-µm area of the sample by the chip size of the CCD camera (Retiga-4000R, QImaging Surrey, Surrey, BC, Canada).

#### 2.2.3. Data Acquisition and Analysis

The time-lapse fluorescence and phase contrast images (Figure 1B) were collected and analyzed as previously described [17,18,19] The image analysis software, CellProfiler (Ver. 2.1.1, the Broad Institute of Harvard and MIT, Cambridge, MA, USA), was used to perform batch image processing and to count the number of bacteria in each image [25]. The ratio of fluorescing bacteria (from the fluorescence image) to total bacteria (in the phase contrast image) represented a normalized cell death count for a specific time point. Cell death was monitored as a function of time as well as a function of antibiotic presence in the media [17,18] (Figure 1C). 

### 2.3. Optimization Experiments

#### 2.3.1. Bacterial Strains

All of the optimization experiments were performed with the MSSA strain Sanger 476 used previously [17,18]. Dose–response experiments were performed with both Sanger 476 and the MRSA strain MW2. Finally, optimized conditions were tested with three strains from the original study: MN8 (MSSA), Newman (MSSA), and NRS382 (MRSA) [18]. The experimental conditions for each test described below are summarized in Table 1. 

#### 2.3.2. Baseline Variation: Temperature Control, Component Quality

All experiments were conducted with 0.7 ng/mL lysostaphin, 50 µg/mL oxacillin, and 0.5 µM SYTOX Orange. Baseline experiments were done within the manufacturers’ recommended usage time for the components and without temperature control. In order to test whether the freshness of the media components had an effect on the cell death response, each media component including MH broth, chemical stressor, and fluorescence stain was intentionally tested within a few days of either being made (in the case of the broth) or purchased (in the case of the SYTOX Orange dye or lysostaphin). Temperature control experiments were conducted with a heater installed inside the experimental setup enclosure with external monitoring of the temperature. The temperature within the enclosure was stabilized at least 10 min prior to the start of the flow test. The 60-min cell death value was normalized to the control channel cell death and the initial cell death value [18] and was used to compare data for different conditions. 

#### 2.3.3. Effect of a Chemical Stressor

The effect of increasing the lysostaphin concentration from the previously used 0.7 ng/mL to 1.4 ng/mL was studied relative to the presence of mechanical stress only. The oxacillin concentration was fixed at 50 µg/mL to maximize antibiotic response, and the environmental temperature was set at 35 °C. The control data with and without the chemical stressor were acquired to account for non-antibiotic-related cell death. 

#### 2.3.4. Increased Shear Stress

Prior to conducting increased shear stress experiments with bacteria, the flow cell was verified as able to sustain high shear stresses up to 250 Pa or up to 18 mL/min. The flow rate was fixed at 0.65 mL/min for the baseline shear stress of 9.3 Pa, given the geometry of the channel. Two options were considered to study the impact of increased stress: constant shear and variable shear. A constant 10-fold shear stress increase was achieved by fixing the flow rate at 6.5 mL/min. Variable shear stress was achieved by alternating between the increased and standard flow rates every 10 min. In each experiment, an individual measurement for the high or variable shear was compared to a concurrent baseline flow measurement with and without antibiotic at the same flow conditions. As the maximum syringe volume in the experimental system was 60 mL, and a 390-mL volume for high shear experiments was required, the flow direction was reversed after every 15 mL of dispensed volume. The lysostaphin and oxacillin concentrations were fixed at 0.7 ng/mL and 50 µg/mL, respectively.

#### 2.3.5. Effect of Media Alteration

To establish the effect of salt in the media on the fluorescence response, a four-way diagonal switch valve (IDEX, Inc., Tewksbury, MA, USA) was introduced into the media delivery line, which allowed for the fast switching (within a few tens of seconds) of the media inside of the channel. MH supplemented with sodium chloride (2% NaCl *w*/*v*) and without supplementation were interchanged between two channels during the course of the experiment. Control channels had two media solutions without switching. All media contained oxacillin at 2 µg/mL, lysostaphin at 1.4 ng/mL, and fluorescence stain. The environmental temperature was held at 35 °C.

#### 2.3.6. Cumulative Effect on Susceptibility Testing

MSSA and MRSA were tested as a function of variable oxacillin concentrations between 1 and 16 µg/mL. The concentrations were selected to cover the range between two minimal inhibitory concentrations: Sanger 476 (0.5 µg/mL) and MW2 (>16 µg/mL). The lysostaphin concentration for these experiments was 1.4 ng/mL and the temperature was controlled at 35 °C. Low salt content media (MH) was used for these experiments.

#### 2.3.7. Optimized Conditions Test

Optimized conditions were set to 2 µg/mL oxacillin, 1.4 ng/mL, and environmental temperature of 35 °C. MH media without added salt was used. The conditions were tested on the subset of previously studied MRSA (NRS382) and MSSA (MN8 and Newman) strains [18]. 

### 2.4. Lysostaphin and Salt Influence on Oxacillin MIC Determination

The effect of salt supplementation of the media and the lysostaphin were benchmarked by standard AST methods for determining the minimal inhibitory concentration (MIC) [8]. Briefly, MIC was determined using the broth microdilution method for oxacillin AST for staphylococcal strains. A few colonies of each bacteria sample picked directly from the plate were suspended into the cation-adjusted MH broth with/without 2% NaCl to the OD of 0.3. This OD corresponds to about 3 × 10^8^ CFU/mL of bacteria. Bacteria were further diluted to the concentration of 1 × 10^6^ CFU/mL. 100 µL of the bacterial suspension was mixed with 100 µL of two-fold-diluted antibiotic in 96-well plates. The plates were incubated overnight at 35 °C and examined for the presence of visible growth after 20–24 h. The MIC was defined as the lowest antibiotic concentration showing no visible bacterial growth.

Variations in the experimental conditions for the MIC studies are summarized in Table 2. The lysostaphin MIC was measured as a function of culture media in the absence of oxacillin (Table 2, first row). The oxacillin MIC was also determined as a function of culture media, with or without lysostaphin at 1.4 ng/mL. The ranges for the oxacillin dilution series were adjusted for susceptible (Table 2, second row) and resistant strains (Table 2, third row).

## 3. Results

### 3.1. Baseline Variation

**Figure 2:**
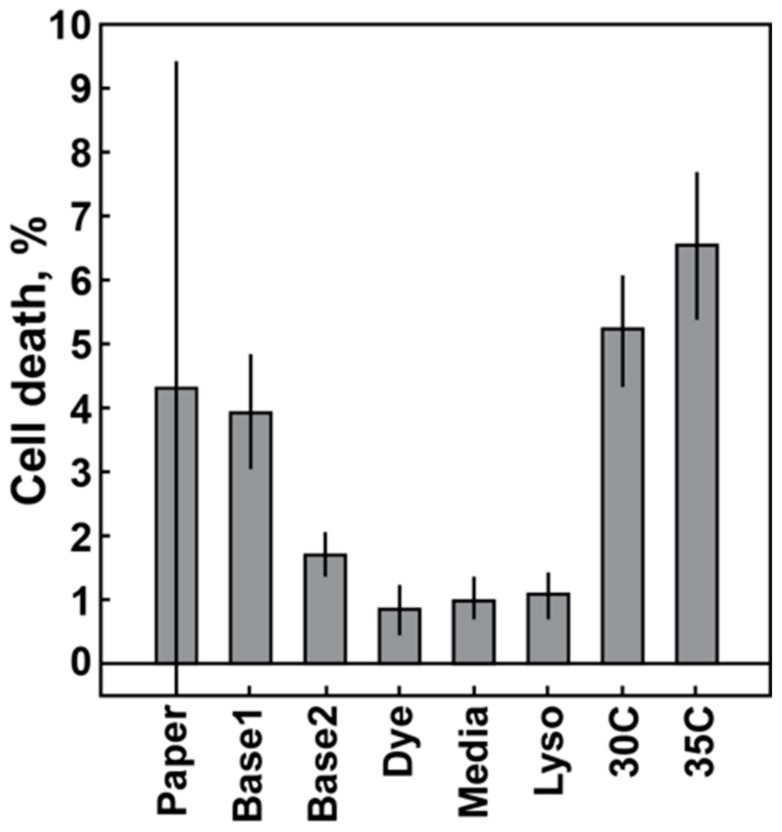
Cell death response of the MSSA Sanger 476 strain at 60 min as a function of the experimental condition. Each column represents an average and standard deviation of multiple replicates for each condition. Column data origin: Paper: data from previously published paper [18]; Base1 and Base2: baseline data acquired during three consecutive weeks with significant change in average response level; Dye: fresh fluorescence stain; Media: fresh cell culture media; Lyso: fresh lysostaphin; 30C, 35C: controlled ambient temperature set value. Fresh: components used within a few days of receipt (for purchased components) or within a few days from production (for in-house components).

Variations in the 60-min cell death response of the MSSA Sanger 476 strain are plotted as a function of different conditions (Figure 2). At least three experimental replicates were completed for each condition. Each column represents an average and a standard deviation of those experiments. The leftmost column depicts the average and standard deviation of the previously published 60-min data for the Sanger 476 response to 50 µg/mL oxacillin of 4.29 ± 3.33% cell death (See Ref. [18], Figure 2 for details). The next two columns (Figure 2, Base1 and Base2) represent sequential data obtained in the span of three consecutive weeks. The data were split into two groups, due to significant differences in the measured average cell death response. The following columns represent experiments with the fresh fluorescent dye (Dye column), fresh media (Media column) and fresh lysostaphin (Lyso column) (see Section 2.3.2). Introducing fresh components did not lead to an increase in average cell death response. The last two columns are results of the controlled temperature experiments with enclosure temperature set to 30 °C and 35 °C (Figure 2, 30C and 35C). Thus, a cell death response equivalent to previously published data was obtained only with increased temperature, whereas the introduction of fresh media components did not have a significant impact on the signal. 

### 3.2. Effect of Chemical Stressor

**Figure 3:**
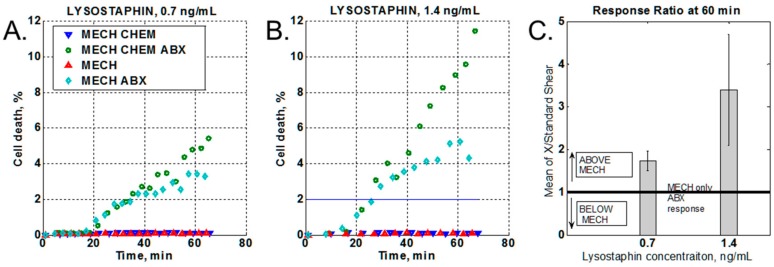
Chemical stressor effect. (**A**,**B**). Time-lapse data of cell death vs time. MECH = mechanical stress (shear flow, no lysostaphin, no oxacillin); ABX = antibiotic (oxacillin at 50 µg/mL); CHEM = chemical stress (lysostaphin at either 0.7 ng/mL or 1.4 ng/mL). (**C**) Ratio of cell death with chemical stressor vs mechanical stress alone.

The presence of a chemical stressor (lysostaphin) produced a marked increase in bacterial cell death (Figure 3A), and an even greater difference when the chemical stressor concentration was increased (Figure 3B). At the same time, the no-antibiotic control shows no response change with increased chemical stressor concentrations, as compared to the no-chemical stressor control (Figure 3B). To compare data from different experiments, the increase in cell death due to the application of the chemical stressor (Figure 3A,B, dark green circles) was normalized to the mechanical-only stress response at 60 min of oxacillin treatment (Figure 3A,B light green rhombus). Thus, values above unity indicated an increase in the bacterial response to the antibiotic in the presence of the chemical stressor as compared to mechanical stress only. The data were statistically averaged over three independent experiments for each lysostaphin concentration (Figure 3C). 

### 3.3. Variation of the Shear Stress

**Figure 4:**
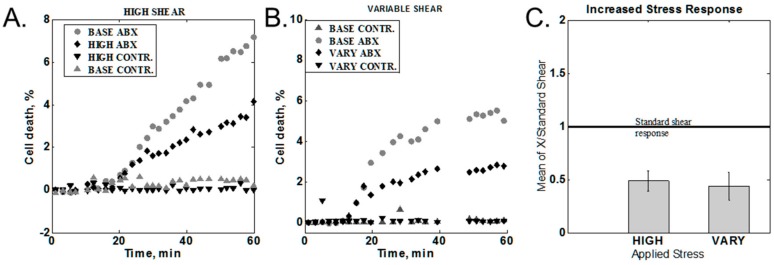
Effect of increased shear response. (**A**) Constant increased shear. BASE = standard shear stress; HIGH = high shear stress (10×), ABX = media with antibiotic, CONTR. = media without antibiotic (**B**) Variable between high and standard shear (1× <-> 10×). VARY = variable shear response. (**C**) Summary of all high-shear measurements.

Shear stress was varied by modulating the flow rate as described in the Section 2.3.4. Two sets of data were obtained: variable and high shear stress. Characteristic behaviors for different cases are shown in Figure 4A,B. In order to compare data from different experiments, the 60-min cell death value of the increased or variable shear was normalized to the 60-min response obtained with standard stress levels. The statistical averages of the responses are summarized in Figure 4C. Ratios below one indicate a lower response for the varied conditions than from standard shear. Both increased and variable shear resulted in a response lower than the standard shear of 9.3 Pa. 

### 3.4. Effect of Media Composition

**Figure 5:**
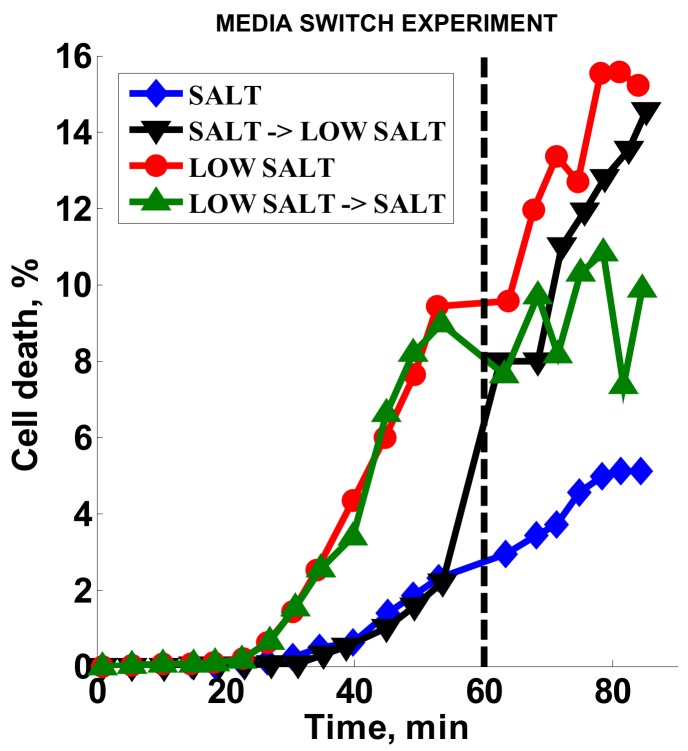
Media salt content effect. Dashed line is the media switch time point. SALT = high-salt media (MHS); LOW SALT = low-salt media (MH); SALT → LOW SALT = switch from high-salt to low-salt media (MHS → MH); LOW SALT → SALT = switch from low-salt to high-salt media (MH → MHS).

Standard AST methods for *S. aureus* and oxacillin call for high salt content media (MHS) [8], which we used to develop our original method. Here, we tested the effect of low-salt media (MH) on the AST method, as well as the response of bacteria when the media is switched from high- to low-salt medium after 60 min (Figure 5). The two channels with MHS (Figure 5, black inverted triangles and blue diamonds) had low cell death (ca. 2%) prior to switching, while the MH channels (Figure 5, red circles and green triangles) showed higher cell death (ca. 10%) during the same timeframe. Over three experiments, the signal obtained with MH was consistently higher than with MHS (ratio of 2.9 ± 0.45). Upon switching from MHS media to MH, the cell death signal increased dramatically (Figure 5, black inverted triangles) almost to the level of the continuous MH signal (Figure 5, red circles). At the same time, the cell death response stopped increasing for the channel where there was a switch from MH to MHS (Figure 5, green triangles). These results indicate that, in our stress-based platform, salt had a dampening effect on the cell death response in the presence of oxacillin.

### 3.5. Cumulative Effect on Susceptibility Testing of Optimized Conditions

**Figure 6:**
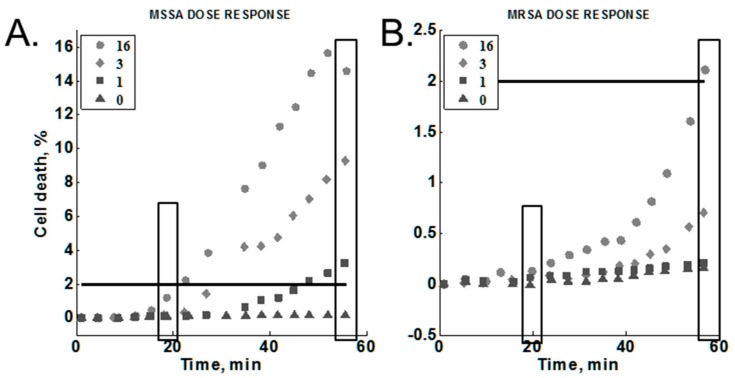
Methicillin-susceptible *Staphylococcus aureus* (MSSA) vs methicillin-susceptible *S. aureus* (MRSA) dose-responses for oxacillin. Legend: antibiotic concentration in µg/mL. 2% black line: provides a relative scale comparison between the two graphs. Rectangles show data selected for Table 3 below.

The concentration of oxacillin was varied to test for a dose-response against MRSA (MW2) and MSSA (Sanger 476) within our platform (Section 2.3.6). The results are summarized in Figure 6 (Note the different *y*-axis scales). These experiments included the optimized lysostaphin concentration (1.4 ng/mL), temperature (35 °C), and media composition (MH; no added salt). We found that cell death increased as a function of time for all antibiotic concentrations tested against the susceptible strain and for all but the lowest concentration tested against the resistant strain. We did observe a dose-response for both of the *S. aureus* strains tested. In each case, the cell death response at a given time point was higher with increasing antibiotic concentrations. All antibiotic concentrations gave greater than 2% cell death for the susceptible strain. In contrast, only the highest antibiotic concentration (16 µg/mL) produced greater than 2% cell death in the resistant strain. 

The signals at 20 min and 60 min were selected to compare end-point and early time point measurements for resistant and susceptible strains (Figure 6, boxes). The susceptible strain response was consistently above the resistant strain response for each concentration at 20 min, reaching a 10:1 difference at the highest antibiotic concentration tested (Table 3, bolded text in bottom row). A greater than 10:1 difference in response was observed for both 1 and 3 µg/mL at 60 min (Table 3, bolded text in second and third rows). Note that the difference at 60 min was greater at the lower antibiotic concentrations. On the other hand, high antibiotic concentrations gave greater ratios at the 20-min time point.

### 3.6. Optimized Conditions Test

**Figure 7:**
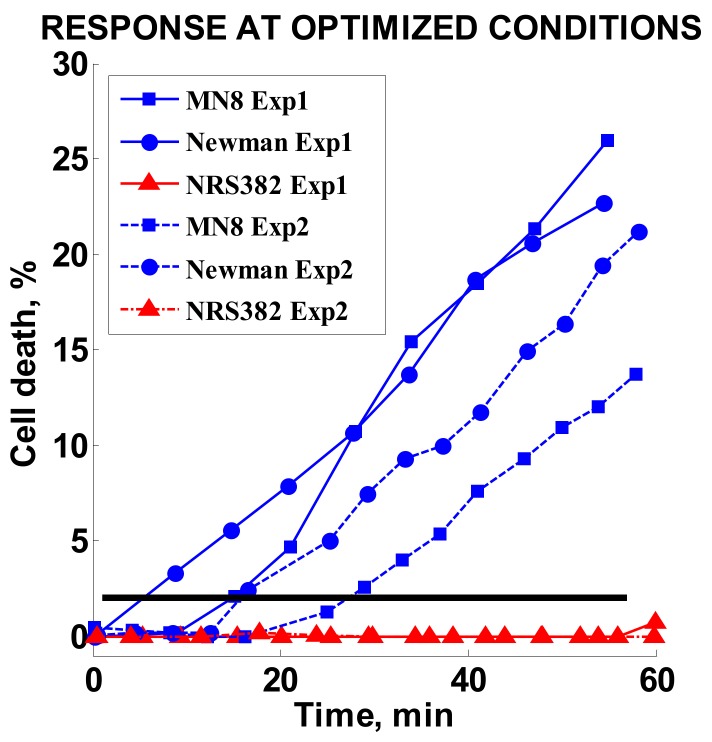
Cell death responses for two independent experiments using optimized conditions. Solid lines denote the first experiment (Exp1) and dashed lines show the second experiment (Exp2). The black horizontal line represents the 2% cell death threshold. Blue symbols were used for MSSA strains and red symbols show minimal death of MRSA strains.

Finally, three strains tested in the original study, MN8 and Newman (MSSA) and MRSA strain NRS382, were retested at optimized conditions. The oxacillin concentration was set to 2 µg/mL. The results of two independent experiments for each strain are summarized in Figure 7. A high response of 13–25% was achieved for the MSSA strains, whereas the MRSA strain response stayed effectively unchanged during the observed time period.

### 3.7. MIC Studies Results

The lysostaphin MIC measured for Sanger 476/MW2 was >250 ng/mL in either salt-supplemented or nonsupplemented media. Therefore, even the highest concentration of lysostaphin of 1.4 ng/mL used in the presented studies was more than 100× below the standard MIC value. We do not expect to see any lysostaphin-only-related cell death.

Results of MIC measurements for oxacillin are summarized in Table 4. Assays performed with or without lysostaphin in MH or MHS broth gave similar MIC values. 

## 4. Discussion and Conclusions

In this paper, we measured the effects of various parameters in our stress-based, microfluidic AST: temperature, variation of mechanical and chemical stress, and media composition. We demonstrate how the use of optimized parameters can increase signal amplitudes and thus increase the sensitivity of our stress-based AST platform. The apparent variability in the susceptible strain response was noted in a prior publication [18]. The consistent drop in the average value of cell death over two different sets of replicate experiments indicated assay sensitivity to a previously uncontrolled experimental parameter (Figure 2, Base1 and Base2). However, the independence of the cell death response on the relative freshness of the media components suggested that the decrease in signal was not due to degradation of key reagents. Rather, it validated the robustness of the method. In contrast, a strong dependence on temperature was observed. This can be naturally attributed to the connection between temperature and metabolic activity of bacteria. It is known that bacteria in less active metabolic states, such as stationary or lag-phases, are less susceptible to antibiotic action [26,27]. Therefore, an increase in average signal levels with an increased temperature seems to be readily explained. Moreover, the elevated temperatures are closer to the physiological conditions experienced by bacteria in the bloodstream. One should note that CLSI (Clinical and Laboratory Standards Institute) does not recommend the testing of MSSA/MRSA resistance to oxacillin above 35 °C due to the possibility of erroneous susceptibility readings [8]. In the original studies, the lab environment did not have temperature controls installed, and room temperature varied between 20 °C and 30 °C on a given experimental day. Only within the controlled environment could the role of the temperature be singled out. 

Although an increase in mechanical and chemical stresses were tested, only chemical stress gave an increase in the susceptible strain response. Even though lysostaphin directly damages the bacterial cell wall [28], the increased lysostaphin concentration was still well below the MIC, which was confirmed by the minimal cell death seen in the no-antibiotic controls (Figure 3A,B, blue triangles) and the MIC results in Table 4. On average, in the presence of oxacillin, the cell death signal obtained with the 1.4 ng/mL chemical stressor concentration was three times greater than the data obtained without any chemical stressor present, while the 0.7 ng/mL chemical stressor concentration provided less than twice as much cell death as mechanical stress + ABX (Figure 3C). Any further optimization of the chemical stressor concentration would need to be monitored against the cell response without antibiotic to ensure that the observed effect is specific to the antibiotic.

The result of the high-shear studies was somewhat unexpected. In both the case of sustained and intermittent high shear, we observed a decrease in cell death as compared to the standard shear control for the same experiment. The most plausible explanation is that reduction in the signal levels is related to the loss of damaged bacteria at higher flow rates. As shear increases, we see a general loss of immobilized bacteria at higher flow rates, reaching about 15% loss for high shear and 18% loss for variable flow relative to the standard flow. The loss is indicative of an overall loss of cells, but in order for cell death counts to drop, there should be a preferential loss of the damaged cell population. The tracking of the individual fluorescing cells may not give enough information if the dead cells are detached from the surface prior to accumulating a sufficient amount of stain for detection. It is theoretically feasible to collect detached cells and measure the proportion of the damaged bacteria in the detached population, and we plan to conduct this experiment in the future. It is also worth noting that in the future, it may be beneficial to reduce the flow rate below our previously chosen standard rate.

Mueller Hinton broth medium for standard susceptibility testing of *S. aureus* to oxacillin contains 2% sodium chloride to mimic physical conditions [8]. This 340-mM NaCl concentration is twice that of the physiological concentration of saline. SYTOX Orange belongs to a class of intercalating-DNA stains [29], and its binding to DNA is sensitive to the ionic strength of the solution and is reduced more than fifty times when salt concentration is changed between 100 mM and 1000 mM [30]. Therefore, in retrospect, it is not surprising to see an increase in fluorescence counts upon removal of the salt from the media (Figure 5). The switch from high salt to low salt results in the immediate increase in the binding of fluorescent molecules. A reverse switch from low- to high-salt media leads to leveling of the fluorescence level rather than an immediate reduction, potentially indicating a slow photobleaching effect for already-bound dye molecules [31].

Comparing optimized microfluidic conditions to the standard microdilution method is an important metric for method validation. Whereas a microfluidic flow cell represents a drastically different environment to standard susceptibility testing conducted either in static microwells or plate cultures, some of the parameters can still be benchmarked. Specifically, the oxacillin MIC was assessed in the presence and absence of lysostaphin, as well as in the presence and absence of media with high salt content (Table 4). Neither variation in the media nor the presence of lysostaphin had an effect on the standard MIC value. Therefore, we can conclude that whatever immediate dynamic variations may take place, the long-term response of the bacterial population remains unaffected.

According to our observed dose-response, it is possible to optimize the susceptibility determination for either fastest detection with increased antibiotic concentration (16 µg/mL at 20 min) or lower antibiotic concentration with longer detection times (1–3 µg/mL at 60 min). Due to the fact that lower antibiotic concentrations are more selective in terms of the amplitude ratio between susceptible and resistant strains and that the clinical breakpoint for *S. aureus* strains is set for oxacillin at 2 µg/mL [8], the lower antibiotic concentrations would be preferable in comparison to conventional testing. 

The modified assay performed under optimized conditions with a subset of previously tested strains at a low antibiotic concentration showed an increase of the signal for susceptible strains while the resistant strain response remained low. In conclusion, we have successfully identified important experimental parameters affecting our rapid AST method for MSSA/MRSA phenotype determination. Parameter optimization resulted in gaining control over the amplitude of the response and time-to-result. In future studies, we plan to expand to a wider array of clinically important antibiotics and bacterial species.

## Figures and Tables

**Table 1:** Summary of experimental conditions (changing variable highlighted with bold text).

Experiment/Parameters	*S. aureus* Strains	Oxacillin, µg/mL	Lysostaphin, ng/mL	Temperature, °C	Flow Rate, mL/min	Media
Baseline variation	Sanger 476	50	0.7	**RT *^a^*, 30, 35**	0.65	MHS *^b^*
Chemical stressor	Sanger 476	50	**0.7, 1.4**	35	0.65	MHS
Increased shear stress	Sanger 476	50	0.7	RT	**0.65, 6.5**	MHS
Media alteration	Sanger 476	2	1.4	35	0.65	**MHS, MH *^c^***
Cumulative effect	Sanger 476, **MW2**	**1, 3, 16**	1.4	35	0.65	MH
Optimized conditions test	**MN8, Newman, NRS382**	2	1.4	35	0.65	MH

*^a^* RT: Room temperature (uncontrolled temperature conditions); *^b^* MHS: Mueller Hinton broth with salt (2% NaCl); *^c^* MH: Mueller Hinton broth.

**Table 2:** Summary of experiments.

Experiment/Parameters	MSSA Strains	MRSA Strains	Oxacillin, µg/mL	Lysostaphin, ng/mL	Media
Lysostaphin MIC	Sanger 476	MW2	0	0–1000	MHS *^b^*, MH *^c^*
Lysostaphin effect on oxacillin MIC	Sanger 476, MN8, Newman		0, 0.125–8 *^a^*	0, 1.4	MHS, MH
Lysostaphin effect on oxacillin MIC		MW2, NRS382	0, 8–256 *^a^*	0, 1.4	MHS, MH

*^a^* 2× serial dilutions of antibiotic; *^b^* MHS: Mueller Hinton broth with salt (2% NaCl); *^c^* MH: Mueller Hinton broth.

**Table 3:** Normalized cell death response of MSSA and MRSA at different time points of exposure to oxacillin.

Oxacillin, µg/mL	20 min			60 min		
	MRSA	MSSA	Ratio *^a^*	MRSA	MSSA	Ratio *^a^*
0	0	0	-	0.15	0.17	1.13
1	0.06	0.11	1.83	0.2	3.26	**16.3** *^b^*
3	0.06	0.23	3.83	0.7	9.3	**13.29** *^b^*
16	0.13	1.5	**11.54** *^b^*	2.1	15.6	7.43

*^a^* Ratio = MSSA response/MRSA response. *^b^* Ratio values above 10 were bolded.

**Table 4:** Oxacillin MIC measurements summary in µg/mL.

Strain/Media Composition	MH	MH + Lyso *^a^*	MHS	MHS + Lyso *^a^*
Sanger 476	0.5	0.5	0.5	0.5–1
MN8	0.25–0.5	0.5	0.25–0.5	0.5
Newman	0.25	0.25	0.5	0.25–0.5
MW2	64	64–128	128	64–128
NRS382	64	64	32	64

*^a^* Lyso: lysostaphin at 1.4 ng/mL.

## References

[1] Marston H.D., Dixon D.M., Knisely J.M., Palmore T.N., Fauci A.S. (2016). Antimicrobial Resistance. JAMA.

[2] National Strategy. https://www.cdc.gov/drugresistance/federal-engagement-in-ar/national-strategy/index.html.

[3] Wood K., Angus D. (2004). Pharmacoeconomic implications of new therapies in sepsis. Pharmacoeconomics.

[4] Kumar A., Roberts D., Wood K.E., Light B., Parrillo J.E., Sharma S., Suppes R., Feinstein D., Zanotti S., Taiberg L. (2006). Duration of hypotension before initiation of effective antimicrobial therapy is the critical determinant of survival in human septic shock. Crit. Care Med..

[5] Chun K., Syndergaard C., Damas C., Trubey R., Mukindaraj A., Qian S., Jin X., Breslow S., Niemz A. (2015). Sepsis Pathogen Identification. J. Lab. Autom..

[6] Sievert D.M., Ricks P., Edwards J.R., Schneider A., Patel J., Srinivasan A., Kallen A., Limbago B., Fridkin S. (2013). Antimicrobial-Resistant Pathogens Associated with Healthcare-Associated Infections Summary of Data Reported to the National Healthcare Safety Network at the Centers for Disease Control and Prevention, 2009–2010. Infect. Control Hosp. Epidemiol..

[7] Centers for Disease Control and Prevention (2016). Biggest Threats/Antibiotic/Antimicrobial Resistance.

[8] Patel J.B. (2015). Performance Standards for Antimicrobial Susceptibility Testing.

[9] Yagupsky P., Nolte F.S. (1990). Quantitative aspects of septicemia. Clin. Microbiol. Rev..

[10] Sabui T., Tudehope D.I., Tilse M. (1999). Clinical significance of quantitative blood cultures in newborn infants. J. Paediatr. Child Health.

[11] Ho T.H., Huang Y.C., Lin T.Y. (2011). Evaluation of the BD GeneOhm StaphSR assay for detection of *Staphylococcus aureus* in patients in intensive care units. J. Microbiol. Immunol. Infect..

[12] Katayama Y., Takeuchi F., Ito T., Ma X.X., Ui-Mizutani Y., Kobayashi I., Hiramatsu K. (2003). Identification in Methicillin-Susceptible Staphylococcus hominis of an Active Primordial Mobile Genetic Element for the Staphylococcal Cassette Chromosome mec of Methicillin-Resistant Staphylococcus aureus. J. Bacteriol..

[13] Köser C.U., Ellington M.J., Peacock S.J. (2014). Whole-genome sequencing to control antimicrobial resistance. Trends Genet..

[14] Suzuki S., Horinouchi T., Furusawa C. (2014). Prediction of antibiotic resistance by gene expression profiles. Nat. Commun..

[15] Campbell J., McBeth C., Kalashnikov M., Boardman A.K., Sharon A., Sauer-Budge A.F. (2016). Microfluidic advances in phenotypic antibiotic susceptibility testing. Biomed. Microdevices.

[16] Choi J., Yoo J., Lee M., Kim E.G., Lee J.S., Lee S., Joo S., Song S.H., Kim E.C., Lee J.C. (2014). A rapid antimicrobial susceptibility test based on single-cell morphological analysis. Sci. Transl. Med..

[17] Kalashnikov M., Campbell J., Lee J.C., Sharon A., Sauer-Budge A.F. (2014). Stress-induced Antibiotic Susceptibility Testing on a Chip. J. Vis. Exp..

[18] Kalashnikov M., Lee J.C., Campbell J., Sharon A., Sauer-Budge A.F. (2012). A microfluidic platform for rapid, stress-induced antibiotic susceptibility testing of Staphylococcus aureus. Lab Chip.

[19] Kalashnikov M., Mueller M., McBeth C., Lee J.C., Campbell J., Sharon A., Sauer-Budge A.F. (2017). Rapid phenotypic stress-based microfluidic antibiotic susceptibility testing of Gram-negative clinical isolates. Sci. Rep..

[20] Santiso R., Tamayo M., Gosálvez J., Bou G., del Carmen Fernández M., Fernández J.L. (2011). A rapid in situ procedure for determination of bacterial susceptibility or resistance to antibiotics that inhibit peptidoglycan biosynthesis. BMC Microbiol..

[21] Huang T.-H., Ning X., Wang X., Murthy N., Tzeng Y.L., Dickson R.M. (2015). Rapid Cytometric Antibiotic Susceptibility Testing Utilizing Adaptive Multidimensional Statistical Metrics. Anal. Chem..

[22] Nonejuie P., Burkart M., Pogliano K., Pogliano J. (2013). Bacterial cytological profiling rapidly identifies the cellular pathways targeted by antibacterial molecules. Proc. Natl. Acad. Sci. USA.

[23] Longo G., Alonso-Sarduy L., Rio L.M., Bizzini A., Trampuz A., Notz J., Dietler G., Kasas S. (2013). Rapid detection of bacterial resistance to antibiotics using AFM cantilevers as nanomechanical sensors. Nat. Nanotechnol..

[24] McDonald J.C., Whitesides G.M. (2002). Poly(dimethylsiloxane) as a material for fabricating microfluidic devices. Acc. Chem. Res..

[25] Carpenter A.E., Jones T.R., Lamprecht M.R., Clarke C., Kang I.H., Friman O., Guertin D.A., Chang J.H., Lindquist R.A., Moffat J. (2006). CellProfiler: Image analysis software for identifying and quantifying cell phenotypes. Genome Biol..

[26] Eng R.H., Padberg F.T., Smith S.M., Tan E.N., Cherubin C.E. (1991). Bactericidal effects of antibiotics on slowly growing and nongrowing bacteria. Antimicrob. Agents Chemother..

[27] Mascio C.T.M., Alder J.D., Silverman J.A. (2007). Bactericidal Action of Daptomycin against Stationary-Phase and Nondividing *Staphylococcus aureus* Cells. Antimicrob. Agents Chemother..

[28] Francius G., Domenech O., Mingeot-Leclercq M.P., Dufrene Y.F. (2008). Direct Observation of *Staphylococcus aureus* Cell Wall Digestion by Lysostaphin. J. Bacteriol..

[29] Armitage B.A. (2005). DNA Binders and Related Subjects.

[30] Biebricher A.S., Heller I., Roijmans R.F., Hoekstra T.P., Peterman E.J., Wuite G.J. (2015). The impact of DNA intercalators on DNA and DNA-processing enzymes elucidated through force-dependent binding kinetics. Nat. Commun..

[31] Thakur S., Cattoni D.I., Nöllmann M. (2015). The fluorescence properties and binding mechanism of SYTOX green, a bright, low photo-damage DNA intercalating agent. Eur. Biophys. J..

